# Automated detection of regions of interest for tissue microarray experiments: an image texture analysis

**DOI:** 10.1186/1471-2342-7-2

**Published:** 2007-03-09

**Authors:** Bilge Karaçali, Aydin Tözeren

**Affiliations:** 1 Center for Integrated Bioinformatics, School of Biomedical Engineering, Science and Health Systems, Drexel University, 3141 Chestnut Street, Philadelphia, Pennsylvania 19104-2875, USA

## Abstract

**Background:**

Recent research with tissue microarrays led to a rapid progress toward quantifying the expressions of large sets of biomarkers in normal and diseased tissue. However, standard procedures for sampling tissue for molecular profiling have not yet been established.

**Methods:**

This study presents a high throughput analysis of texture heterogeneity on breast tissue images for the purpose of identifying regions of interest in the tissue for molecular profiling via tissue microarray technology. Image texture of breast histology slides was described in terms of three parameters: the percentage of area occupied in an image block by chromatin (*B*), percentage occupied by stroma-like regions (*P*), and a statistical heterogeneity index *H *commonly used in image analysis. Texture parameters were defined and computed for each of the thousands of image blocks in our dataset using both the gray scale and color segmentation. The image blocks were then classified into three categories using the texture feature parameters in a novel statistical learning algorithm. These categories are as follows: image blocks specific to normal breast tissue, blocks specific to cancerous tissue, and those image blocks that are non-specific to normal and disease states.

**Results:**

Gray scale and color segmentation techniques led to identification of same regions in histology slides as cancer-specific. Moreover the image blocks identified as cancer-specific belonged to those cell crowded regions in whole section image slides that were marked by two pathologists as regions of interest for further histological studies.

**Conclusion:**

These results indicate the high efficiency of our automated method for identifying pathologic regions of interest on histology slides. Automation of critical region identification will help minimize the inter-rater variability among different raters (pathologists) as hundreds of tumors that are used to develop an array have typically been evaluated (graded) by different pathologists. The region of interest information gathered from the whole section images will guide the excision of tissue for constructing tissue microarrays and for high throughput profiling of global gene expression.

## 1 Background

The standard procedure in clinical assessment of invasive breast cancer is the classification of the tumor into one of the three distinct histology grades [1]. Main difficulties related to grading of breast cancer in a reliable and reproducible fashion have been attributed by researchers to the arbitrary mathematical formula for grade assignment, observer-dependent evaluation of the grade parameters and the cellular and texture heterogeneity of the tumor [2,3]. Recent advances in global gene expression profiling and tissue microarrays have uncovered the potential of biomarker expression sets in clinically relevant classification and subsequent individualized treatment [4-9].

Rapid progress is being made in developing gene chips with high diagnostic potential [10-12]. Similarly, recent advances in the development of high density tissue microarrays allow the assessment of multiple protein expression for diagnostic and prognostic purposes over a large number of tissue sections from breast disease tissue banks [13-16]. Both the gene chip and tissue microarray methods require sampling of the tumor tissue at a location containing large amounts of cancer cells. Because these methods are so new, standard automated protocols have not yet been developed to identify the regions of interest in the tumor tissue. Instead, these regions are selected on the basis of the visual evaluation of histology slide images by expert pathologists and as such, the molecular profiling obtained for the tissue with these new high throughput methods may be operator dependent. Present study aims to develop automated procedures for identifying cancer cell rich regions of interest in whole section histology slides for guidance in sampling tissue in constructing tissue microarrays. Our automated image processing method is capable of classifying breast histology image blocks into three clusters specific to normal appearance, specific to cancerous appearance, and those that are not specific to either. The spatial distributions of cancer-specific image blocks predicted using the statistical learning algorithms developed in this study can be used to guide the sampling of tumor tissue for constructing tissue microarrays. Advanced image analysis such as those that are present in the literature can then be utilized to process the biomarker decorated images in tissue microarrays for clinically relevant classification of the tissue [1,17-26].

## 2 Methods

**Figure 1:**
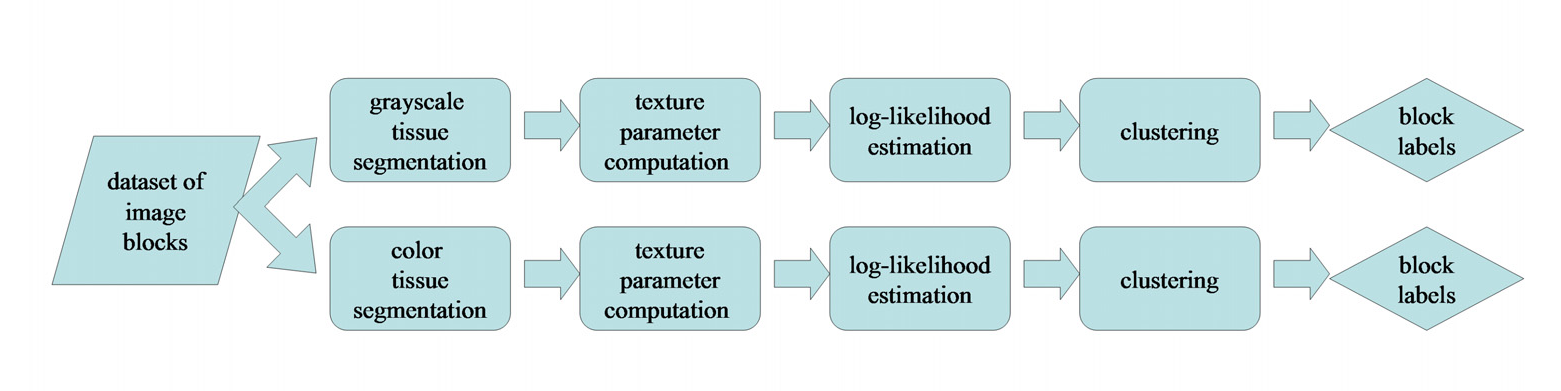
Flowchart of the methods used to analyze image blocks and determine the normal-specific, cancer-specific, and non-specific clusters.

In this section, we describe our dataset of image blocks, the series of automated image analysis algorithms that were used to collect image texture parameters for these blocks, and the statistical learning algorithm developed in this study to classify these blocks into normal-specific, cancer-specific, and non-specific categories. A flowchart of the methodology is shown in Figure 1. The details on each step are described below.

### 2.1 Histology image dataset

**Figure 2:**
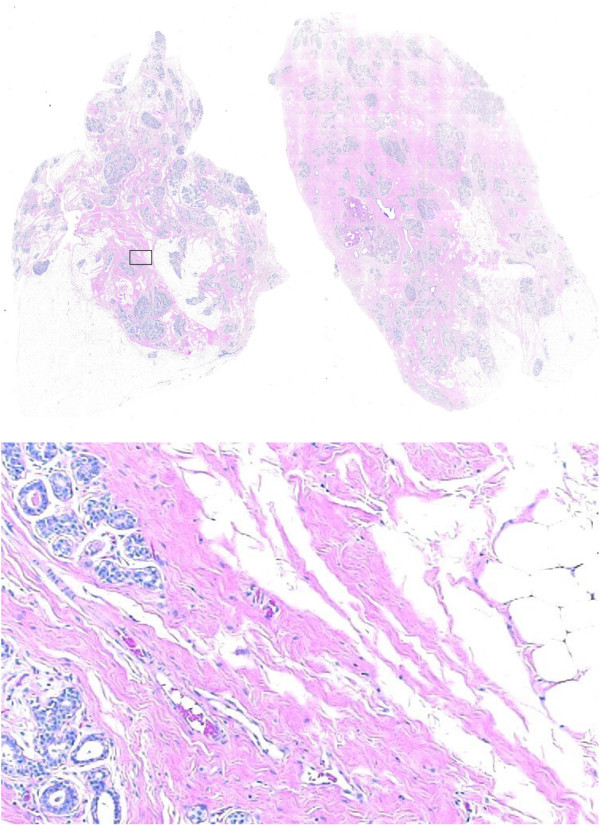
A histology slide of benign breast tissue stained with H&E and an image spot of size comparable to the tissue samples used in tissue microarrays. The image block is 0.66 × 1.03 *mm*^2 ^while the full tissue section is 2.04 × 2.89 *cm*^2^.

The image dataset used in this analysis was obtained by capturing the digital images of 14 Hematoxylin and Eosin (H&E) stained whole section breast tissue slides from a total of 6 specimens. This collection of histology slides was provided to this study by Dr. Jeffrey Hooke of Walter Reed Army Medical Center. Drexel University Institutional Review Board reviewed our research concerning histology slides taken from breast tumors from patients whose identities were undisclosed, and determined that it was in compliance with Federal-Wide Assurance # 00001852 on the treatment of human subjects as well as being in compliance with Drexel University research policy involving biological samples with undisclosed private information. The images were taken using a Nikon Coolscope VS digital microscope (Nikon Corporation Co., Ltd., Parale Mitsui Bldg., 8, Higashida-cho, Kawasaki-ku, Kawasaki, Kanagawa, 210-0005, Japan) at 10× magnification, corresponding to a pixel size of approximately 1.37 *μm *× 1.37 *μm*. The digitization of whole section slides was achieved in terms of consecutive frames (7817 overall for all 14 histology slides) that reconstruct a complete slide when put together in the proper order. Among the 14 slides, 5 have been determined to exhibit normal or benign appearances. *Invasive Ductal Carcinoma *(IDC) have been identified in the remaining 9 slides both by Dr. Jeffrey Hooke and Dr. Min Huang of Fox Chase Cancer Center. An example of an image bock of a benign breast histology slide is shown in Figure 2 along with a constituent frame. Note that the image frame is a rectangle with dimensions 0.66 × 1.03 *mm*^2^. The size of the rectangle is comparable to the size of blocks used in tissue microarrays [15,16]. The typical dimensions of the whole section slides used in this study are 2.04 × 2.89 *cm*^2^.

### 2.2 Adaptive image segmentation

Histology slides of breast tissue stained with H&E show chromatin-rich regions in blue, the surrounding stroma in pink, and the unstained regions in white. In this study, we have implemented grayscale and color-based segmentation algorithms to partition histology images into three primary regions: chromatin-rich, stromal tissue, and the unstained regions. These segmentation algorithms are described below.

#### 2.2.1 Grayscale segmentation

**Figure 3:**
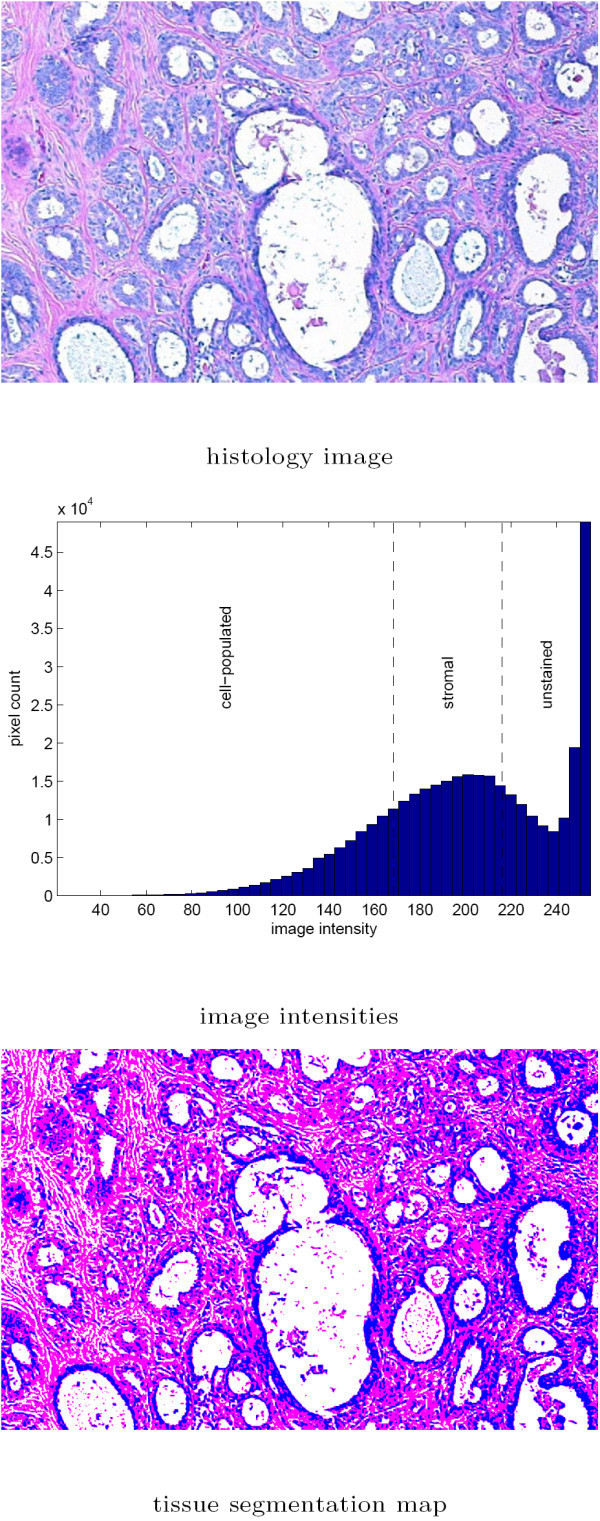
Histology image segmentation into chromatin-rich, stromal, and unstained regions using image intensity. A grayscale value is computed for each pixel as the average of red, green, and blue color channels, and a 3 cluster *k*-means algorithm is used to determine the intensity thresholds between the respective clusters. The lowest intensity range is associated with the cell populated regions, while the highest intensity range determines the adipose tissue and the background. The middle intensity range corresponds to the stroma.

The image intensity observed in each pixel of a given image was first expressed as the average of red, green, and blue color channels. Then a *k*-means unsupervised clustering algorithm [27,28] was carried out to identify the intensity thresholds between the darker chromatin-rich regions, relatively brighter stroma regions, and the brightest unstained regions. For improved convergence and stability, the pixels with saturated intensity were excluded from the segmentation. The *k*-means algorithm was initialized at the smallest, median, and largest intensity values. The grayscale segmentation process used in the study is illustrated in Figure 3.

#### 2.2.2 Color segmentation

The first step in color segmentation was to compute the image representation in the CIE *Lab *color space [29] via conversion from the *RGB *color space used by most image acquisition devices. The *Lab *representation carries several advantages over the *RGB *space [30]: First and foremost, it allows constructing a device-independent representation of the color scene by factoring in the color of the ambient light and constructing unbiased luminance and chromaticity indices. Secondly, it approximates the color differences perceived by an average human in the computed color indices, so that a more uniform distribution is obtained among similar colors.

Adaptive segmentation of histology images into their constituents were implemented in two successive steps. First, we used the luminance index to determine the foreground (consisting of chromatin-rich and stromal regions) from the background (unstained regions). For this purpose, an iterative algorithm was used to fit a two-component Weibull mixture to the observed luminance indices. The algorithm performed repeated line searches to find the mixture parameters that optimized the fit to the observed values. The maximum likelihood threshold between the two components then determined the luminance threshold that separated the chromatin-rich and stromal regions from the unstained regions.

**Figure 4:**
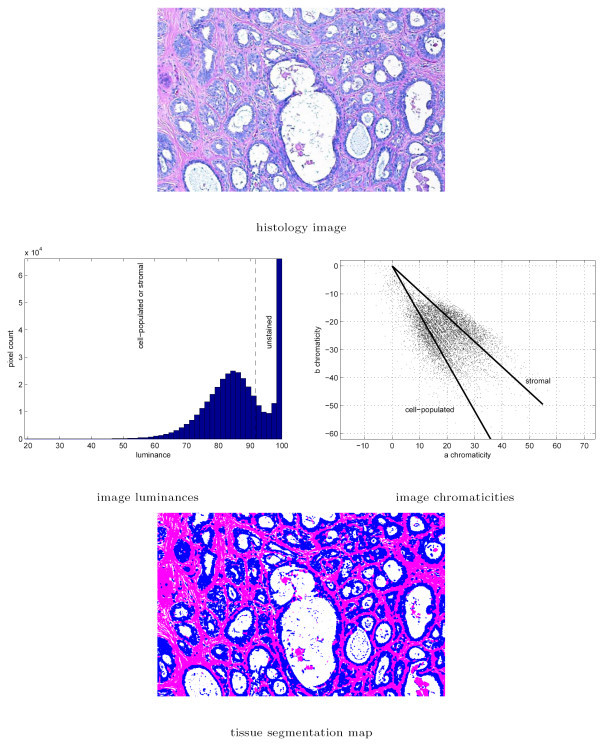
Histology image segmentation into chromatin-rich, stromal, and unstained regions using color information. At the first step, unstained regions are separated from the chromatin-rich and stromal regions based on the luminance indices. The chromatin-rich and stromal regions are identified in the second step using a modified 2-cluster *k*-means algorithm on the chromaticity indices.

In the next step, we used a modified *k*-means clustering algorithm to separate chromatin-rich regions from the stroma. Relative magnitudes of the pixel chromaticity indices were utilized as guide for the desired separation. By virtue of the *Lab *color space, the pixels of chromatin-rich and stromal regions in H&E stained histology slides accumulated around distinct vectors in the *a *and *b *chromaticity space centered at the origin. We computed these chromaticity vectors by defining cluster centers as directional vectors that minimized the average Euclidean distance from the pixel chromaticity indices to their respective cluster centers in a *k*-means iteration. The full color segmentation algorithm used in this study is illustrated in Figure 4.

### 2.3 Texture parameters of segmented histology image blocks

The texture properties of each image block were represented in the present study by three parameters: the percentage of area of the image covered by chromatin-rich cell nuclei (*B*), percentage occupied by collagen-rich stroma (*P*), and a parameter of spatial heterogeneity represented in this study as *H*. We have defined *H *as the probability of a pair of neighboring pixels to belong to different tissue constituents among all possible pairs observed in the image. The parameter H is lower in image blocks where different tissue constituents are aggregated together in comparison to image blocks where the constituents are dispersed among and across each other. All three of these parameters vary between 0 and 1. Moreover, the percentage of unstained area in an image block denoted by *W *then satisfies *W *= 1 - *B *- *P*.

The texture parameters were computed using grayscale and color segmentation algorithms for each image block in the dataset:

*MP*_*grayscale *_= [*B*_*grayscale *_*P*_*grayscale *_*H*_*grayscale*_]^*T*^

and

*MP*_*color *_= [*B*_*color *_*P*_*color *_*H*_*color*_]^*T*^

These texture parameters were used for classification within their respective cohorts. The texture profiles associated with the image block in Figures 3 and 4 are *MP*_*grayscale *_= [0.2105 0.4108 0.2397]^*T *^and *MP*_*color *_= [0.3682 0.3233 0.1176]^*T *^based on the tissue segmentation maps obtained using the two segmentation methods.

**Figure 5:**
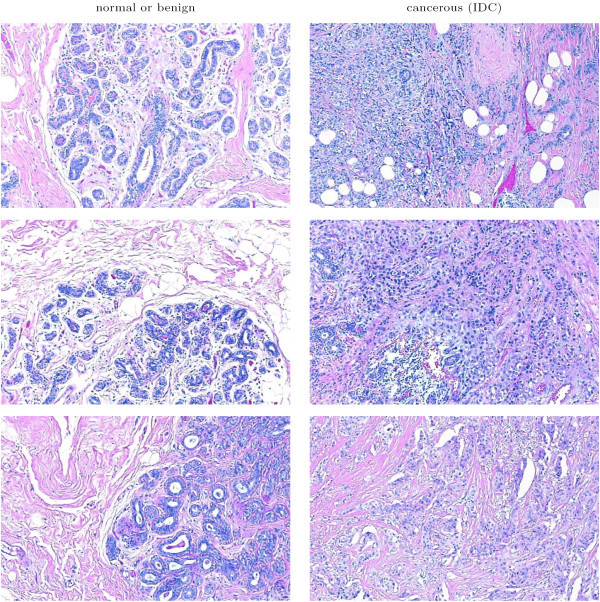
Randomly selected image blocks from the ground truth dataset. The images on the left indicate normal/benign breast tissue, while the images on the right represent examples of Invasive Ductal Carcinoma.

Image blocks that contained extensive fat or unstained region were excluded from our dataset via the use of a ground truth data subset. This subset contained 16 frames of benign presentation and 20 frames of IDC. Randomly selected elements of this subset are shown in Figure 5. The ground truth data subset was used to estimate the upper bound for unstained regions in both cancerous and benign tissue in cell crowded regions. Image blocks that had white in amounts greater than this upper bound were eliminated from the dataset for further analysis. The resulting dataset contained 2395 image blocks for which image texture analysis was conducted.

### 2.4 Statistical learning for detecting cancer-specific tissue image regions

Image blocks in our dataset were classified using the *B*, *P*, and *H *values for each block into three clusters: those observed in normal (**N**) or cancerous specimens (**C**) along with those observed frequently in both tissue types (**G**). To this end, we estimated the log-likelihood ratios of probability density functions that govern the distributions of texture profiles of image blocks obtained from normal and cancerous whole section slides. This estimation was performed using a nonparametric method at each image block described below.

Let *p*_*n *_and *p*_*c *_denote the probability density functions for the texture profiles of image blocks observed in normal and cancerous specimens. We defined the normal-specific, cancer-specific, and non-specific image block clusters based on their texture profiles as

CN={MP|log⁡(pn(MP)pc(MP))>τ}     (1)
 MathType@MTEF@5@5@+=feaafiart1ev1aaatCvAUfKttLearuWrP9MDH5MBPbIqV92AaeXatLxBI9gBaebbnrfifHhDYfgasaacH8akY=wiFfYdH8Gipec8Eeeu0xXdbba9frFj0=OqFfea0dXdd9vqai=hGuQ8kuc9pgc9s8qqaq=dirpe0xb9q8qiLsFr0=vr0=vr0dc8meaabaqaciaacaGaaeqabaqabeGadaaakeaat0uy0HwzTfgDPnwy1egaryqtHrhAL1wy0L2yHvdaiqaacqWFce=qdaWgaaWcbaacbeGae4Nta4eabeaakiabg2da9maacmqabaGaemyta0Kaemiuaa1aaqqaaeaacyGGSbaBcqGGVbWBcqGGNbWzdaqadaqaamaalaaabaGaemiCaa3aaSbaaSqaaiabd6gaUbqabaGccqGGOaakcqWGnbqtcqWGqbaucqGGPaqkaeaacqWGWbaCdaWgaaWcbaGaem4yamgabeaakiabcIcaOiabd2eanjabdcfaqjabcMcaPaaaaiaawIcacaGLPaaacqGH+aGpiiGacqqFepaDaiaawEa7aaGaay5Eaiaaw2haaiaaxMaacaWLjaWaaeWaaeaacqaIXaqmaiaawIcacaGLPaaaaaa@5AD0@

CC={MP|log⁡(pn(MP)pc(MP))<−τ}     (2)
 MathType@MTEF@5@5@+=feaafiart1ev1aaatCvAUfKttLearuWrP9MDH5MBPbIqV92AaeXatLxBI9gBaebbnrfifHhDYfgasaacH8akY=wiFfYdH8Gipec8Eeeu0xXdbba9frFj0=OqFfea0dXdd9vqai=hGuQ8kuc9pgc9s8qqaq=dirpe0xb9q8qiLsFr0=vr0=vr0dc8meaabaqaciaacaGaaeqabaqabeGadaaakeaat0uy0HwzTfgDPnwy1egaryqtHrhAL1wy0L2yHvdaiqaacqWFce=qdaWgaaWcbaacbeGae43qameabeaakiabg2da9maacmqabaGaemyta0Kaemiuaa1aaqqaaeaacyGGSbaBcqGGVbWBcqGGNbWzdaqadaqaamaalaaabaGaemiCaa3aaSbaaSqaaiabd6gaUbqabaGccqGGOaakcqWGnbqtcqWGqbaucqGGPaqkaeaacqWGWbaCdaWgaaWcbaGaem4yamgabeaakiabcIcaOiabd2eanjabdcfaqjabcMcaPaaaaiaawIcacaGLPaaacqGH8aapcqGHsisliiGacqqFepaDaiaawEa7aaGaay5Eaiaaw2haaiaaxMaacaWLjaWaaeWaaeaacqaIYaGmaiaawIcacaGLPaaaaaa@5BA5@

CG={MP|log⁡(pn(MP)pc(MP))≤τ and log⁡(pn(MP)pc(MP))≥−τ}     (3)
 MathType@MTEF@5@5@+=feaafiart1ev1aaatCvAUfKttLearuWrP9MDH5MBPbIqV92AaeXatLxBI9gBaebbnrfifHhDYfgasaacH8akY=wiFfYdH8Gipec8Eeeu0xXdbba9frFj0=OqFfea0dXdd9vqai=hGuQ8kuc9pgc9s8qqaq=dirpe0xb9q8qiLsFr0=vr0=vr0dc8meaabaqaciaacaGaaeqabaqabeGadaaakeaat0uy0HwzTfgDPnwy1egaryqtHrhAL1wy0L2yHvdaiqaacqWFce=qdaWgaaWcbaacbeGae43raCeabeaakiabg2da9maacmqabaGaemyta0Kaemiuaa1aaqqaaeaacyGGSbaBcqGGVbWBcqGGNbWzdaqadaqaamaalaaabaGaemiCaa3aaSbaaSqaaiabd6gaUbqabaGccqGGOaakcqWGnbqtcqWGqbaucqGGPaqkaeaacqWGWbaCdaWgaaWcbaGaem4yamgabeaakiabcIcaOiabd2eanjabdcfaqjabcMcaPaaaaiaawIcacaGLPaaacqGHKjYOiiGacqqFepaDaiaawEa7aiabbccaGiabbggaHjabb6gaUjabbsgaKjabbccaGiGbcYgaSjabc+gaVjabcEgaNnaabmaabaWaaSaaaeaacqWGWbaCdaWgaaWcbaGaemOBa4gabeaakiabcIcaOiabd2eanjabdcfaqjabcMcaPaqaaiabdchaWnaaBaaaleaacqWGJbWyaeqaaOGaeiikaGIaemyta0KaemiuaaLaeiykaKcaaaGaayjkaiaawMcaaiabgwMiZkabgkHiTiab9r8a0bGaay5Eaiaaw2haaiaaxMaacaWLjaWaaeWaaeaacqaIZaWmaiaawIcacaGLPaaaaaa@7912@

where C
 MathType@MTEF@5@5@+=feaafiart1ev1aaatCvAUfKttLearuWrP9MDH5MBPbIqV92AaeXatLxBI9gBaebbnrfifHhDYfgasaacH8akY=wiFfYdH8Gipec8Eeeu0xXdbba9frFj0=OqFfea0dXdd9vqai=hGuQ8kuc9pgc9s8qqaq=dirpe0xb9q8qiLsFr0=vr0=vr0dc8meaabaqaciaacaGaaeqabaqabeGadaaakeaat0uy0HwzTfgDPnwy1egaryqtHrhAL1wy0L2yHvdaiqaacqWFce=qaaa@3824@_**N**_, C
 MathType@MTEF@5@5@+=feaafiart1ev1aaatCvAUfKttLearuWrP9MDH5MBPbIqV92AaeXatLxBI9gBaebbnrfifHhDYfgasaacH8akY=wiFfYdH8Gipec8Eeeu0xXdbba9frFj0=OqFfea0dXdd9vqai=hGuQ8kuc9pgc9s8qqaq=dirpe0xb9q8qiLsFr0=vr0=vr0dc8meaabaqaciaacaGaaeqabaqabeGadaaakeaat0uy0HwzTfgDPnwy1egaryqtHrhAL1wy0L2yHvdaiqaacqWFce=qaaa@3824@_**C**_, and C
 MathType@MTEF@5@5@+=feaafiart1ev1aaatCvAUfKttLearuWrP9MDH5MBPbIqV92AaeXatLxBI9gBaebbnrfifHhDYfgasaacH8akY=wiFfYdH8Gipec8Eeeu0xXdbba9frFj0=OqFfea0dXdd9vqai=hGuQ8kuc9pgc9s8qqaq=dirpe0xb9q8qiLsFr0=vr0=vr0dc8meaabaqaciaacaGaaeqabaqabeGadaaakeaat0uy0HwzTfgDPnwy1egaryqtHrhAL1wy0L2yHvdaiqaacqWFce=qaaa@3824@_**G **_denote the three clusters, and *τ *= log(95/5) is the 95% specificity threshold that ensures that no more than 5% of image blocks assigned to normal-specific and cancer-specific clusters are mislabeled.

We estimated the likelihood ratio *p*_*n *_(*MP*)/*p*_*c *_(*MP*) for a texture profile *MP *using an asymptotic property of a nearest neighbor classification rule. Suppose a texture profile *MP *is given, and a series of nearest neighbor classifiers have been invoked using randomly selected reference datasets with *N *samples observed in normal slides and *N *samples observed from cancerous slides. Suppose also that out of a total of *M *such classifications, the profile *MP *is assigned to the normal class *M*_*n *_times and to the cancerous class *M*_*c *_= *M *- *M*_*n *_times. It can be shown that for large *N *and large *M*, *p*_*n *_(*MP*)/*p*_*c *_(*MP*) ≃ *M*_*n*_/*M*_*c*_.

Let O
 MathType@MTEF@5@5@+=feaafiart1ev1aaatCvAUfKttLearuWrP9MDH5MBPbIqV92AaeXatLxBI9gBaebbnrfifHhDYfgasaacH8akY=wiFfYdH8Gipec8Eeeu0xXdbba9frFj0=OqFfea0dXdd9vqai=hGuQ8kuc9pgc9s8qqaq=dirpe0xb9q8qiLsFr0=vr0=vr0dc8meaabaqaciaacaGaaeqabaqabeGadaaakeaat0uy0HwzTfgDPnwy1egaryqtHrhAL1wy0L2yHvdaiqaacqWFoe=taaa@383C@_*n *_and O
 MathType@MTEF@5@5@+=feaafiart1ev1aaatCvAUfKttLearuWrP9MDH5MBPbIqV92AaeXatLxBI9gBaebbnrfifHhDYfgasaacH8akY=wiFfYdH8Gipec8Eeeu0xXdbba9frFj0=OqFfea0dXdd9vqai=hGuQ8kuc9pgc9s8qqaq=dirpe0xb9q8qiLsFr0=vr0=vr0dc8meaabaqaciaacaGaaeqabaqabeGadaaakeaat0uy0HwzTfgDPnwy1egaryqtHrhAL1wy0L2yHvdaiqaacqWFoe=taaa@383C@_*c *_represent the classes of texture profiles collected from normal and cancerous histology slides respectively. Given the texture profiles {*MP*_*i*_}, *i *= 1, ..., ℓ, collected from image blocks observed in normal and cancerous histology slides, we first normalized the corresponding texture parameters observed across the dataset so that they span the range [0, 1] as uniformly as possible, and then used the algorithm below to estimate their likelihood ratios.

• initialize Mni
 MathType@MTEF@5@5@+=feaafiart1ev1aaatCvAUfKttLearuWrP9MDH5MBPbIqV92AaeXatLxBI9gBaebbnrfifHhDYfgasaacH8akY=wiFfYdH8Gipec8Eeeu0xXdbba9frFj0=OqFfea0dXdd9vqai=hGuQ8kuc9pgc9s8qqaq=dirpe0xb9q8qiLsFr0=vr0=vr0dc8meaabaqaciaacaGaaeqabaqabeGadaaakeaacqWGnbqtdaqhaaWcbaGaemOBa4gabaGaemyAaKgaaaaa@30BC@ = 0 and Mci
 MathType@MTEF@5@5@+=feaafiart1ev1aaatCvAUfKttLearuWrP9MDH5MBPbIqV92AaeXatLxBI9gBaebbnrfifHhDYfgasaacH8akY=wiFfYdH8Gipec8Eeeu0xXdbba9frFj0=OqFfea0dXdd9vqai=hGuQ8kuc9pgc9s8qqaq=dirpe0xb9q8qiLsFr0=vr0=vr0dc8meaabaqaciaacaGaaeqabaqabeGadaaakeaacqWGnbqtdaqhaaWcbaGaem4yamgabaGaemyAaKgaaaaa@30A6@ = 0 for all *i *= 1, ..., ℓ

• for *j *= 1, ..., *M *do

- randomly select *N *texture profiles of image blocks observed in normal histology slides and *N *profiles from those observed in cancerous slides

- collect all selected profiles into a reference dataset

- assign each texture profile to O
 MathType@MTEF@5@5@+=feaafiart1ev1aaatCvAUfKttLearuWrP9MDH5MBPbIqV92AaeXatLxBI9gBaebbnrfifHhDYfgasaacH8akY=wiFfYdH8Gipec8Eeeu0xXdbba9frFj0=OqFfea0dXdd9vqai=hGuQ8kuc9pgc9s8qqaq=dirpe0xb9q8qiLsFr0=vr0=vr0dc8meaabaqaciaacaGaaeqabaqabeGadaaakeaat0uy0HwzTfgDPnwy1egaryqtHrhAL1wy0L2yHvdaiqaacqWFoe=taaa@383C@_*n *_or O
 MathType@MTEF@5@5@+=feaafiart1ev1aaatCvAUfKttLearuWrP9MDH5MBPbIqV92AaeXatLxBI9gBaebbnrfifHhDYfgasaacH8akY=wiFfYdH8Gipec8Eeeu0xXdbba9frFj0=OqFfea0dXdd9vqai=hGuQ8kuc9pgc9s8qqaq=dirpe0xb9q8qiLsFr0=vr0=vr0dc8meaabaqaciaacaGaaeqabaqabeGadaaakeaat0uy0HwzTfgDPnwy1egaryqtHrhAL1wy0L2yHvdaiqaacqWFoe=taaa@383C@_*c *_using a nearest neighbor rule based on the reference dataset

- for *i *= 1, ..., ℓ do

* if *MP*_*i *_is not in the reference dataset, then

• if *MP*_*i *_is assigned to O
 MathType@MTEF@5@5@+=feaafiart1ev1aaatCvAUfKttLearuWrP9MDH5MBPbIqV92AaeXatLxBI9gBaebbnrfifHhDYfgasaacH8akY=wiFfYdH8Gipec8Eeeu0xXdbba9frFj0=OqFfea0dXdd9vqai=hGuQ8kuc9pgc9s8qqaq=dirpe0xb9q8qiLsFr0=vr0=vr0dc8meaabaqaciaacaGaaeqabaqabeGadaaakeaat0uy0HwzTfgDPnwy1egaryqtHrhAL1wy0L2yHvdaiqaacqWFoe=taaa@383C@_*n*_, increment Mni
 MathType@MTEF@5@5@+=feaafiart1ev1aaatCvAUfKttLearuWrP9MDH5MBPbIqV92AaeXatLxBI9gBaebbnrfifHhDYfgasaacH8akY=wiFfYdH8Gipec8Eeeu0xXdbba9frFj0=OqFfea0dXdd9vqai=hGuQ8kuc9pgc9s8qqaq=dirpe0xb9q8qiLsFr0=vr0=vr0dc8meaabaqaciaacaGaaeqabaqabeGadaaakeaacqWGnbqtdaqhaaWcbaGaemOBa4gabaGaemyAaKgaaaaa@30BC@ ← Mni
 MathType@MTEF@5@5@+=feaafiart1ev1aaatCvAUfKttLearuWrP9MDH5MBPbIqV92AaeXatLxBI9gBaebbnrfifHhDYfgasaacH8akY=wiFfYdH8Gipec8Eeeu0xXdbba9frFj0=OqFfea0dXdd9vqai=hGuQ8kuc9pgc9s8qqaq=dirpe0xb9q8qiLsFr0=vr0=vr0dc8meaabaqaciaacaGaaeqabaqabeGadaaakeaacqWGnbqtdaqhaaWcbaGaemOBa4gabaGaemyAaKgaaaaa@30BC@ + 1

• if *MP*_*i *_is assigned to O
 MathType@MTEF@5@5@+=feaafiart1ev1aaatCvAUfKttLearuWrP9MDH5MBPbIqV92AaeXatLxBI9gBaebbnrfifHhDYfgasaacH8akY=wiFfYdH8Gipec8Eeeu0xXdbba9frFj0=OqFfea0dXdd9vqai=hGuQ8kuc9pgc9s8qqaq=dirpe0xb9q8qiLsFr0=vr0=vr0dc8meaabaqaciaacaGaaeqabaqabeGadaaakeaat0uy0HwzTfgDPnwy1egaryqtHrhAL1wy0L2yHvdaiqaacqWFoe=taaa@383C@_*c*_, increment Mci
 MathType@MTEF@5@5@+=feaafiart1ev1aaatCvAUfKttLearuWrP9MDH5MBPbIqV92AaeXatLxBI9gBaebbnrfifHhDYfgasaacH8akY=wiFfYdH8Gipec8Eeeu0xXdbba9frFj0=OqFfea0dXdd9vqai=hGuQ8kuc9pgc9s8qqaq=dirpe0xb9q8qiLsFr0=vr0=vr0dc8meaabaqaciaacaGaaeqabaqabeGadaaakeaacqWGnbqtdaqhaaWcbaGaem4yamgabaGaemyAaKgaaaaa@30A6@ ← Mci
 MathType@MTEF@5@5@+=feaafiart1ev1aaatCvAUfKttLearuWrP9MDH5MBPbIqV92AaeXatLxBI9gBaebbnrfifHhDYfgasaacH8akY=wiFfYdH8Gipec8Eeeu0xXdbba9frFj0=OqFfea0dXdd9vqai=hGuQ8kuc9pgc9s8qqaq=dirpe0xb9q8qiLsFr0=vr0=vr0dc8meaabaqaciaacaGaaeqabaqabeGadaaakeaacqWGnbqtdaqhaaWcbaGaem4yamgabaGaemyAaKgaaaaa@30A6@ + 1

• for *i *= 1, ..., ℓ do

- for each texture profile *MP*_*i*_, compute the estimated log-likelihood ratio *LLR*_*i *_= Mni
 MathType@MTEF@5@5@+=feaafiart1ev1aaatCvAUfKttLearuWrP9MDH5MBPbIqV92AaeXatLxBI9gBaebbnrfifHhDYfgasaacH8akY=wiFfYdH8Gipec8Eeeu0xXdbba9frFj0=OqFfea0dXdd9vqai=hGuQ8kuc9pgc9s8qqaq=dirpe0xb9q8qiLsFr0=vr0=vr0dc8meaabaqaciaacaGaaeqabaqabeGadaaakeaacqWGnbqtdaqhaaWcbaGaemOBa4gabaGaemyAaKgaaaaa@30BC@/Mci
 MathType@MTEF@5@5@+=feaafiart1ev1aaatCvAUfKttLearuWrP9MDH5MBPbIqV92AaeXatLxBI9gBaebbnrfifHhDYfgasaacH8akY=wiFfYdH8Gipec8Eeeu0xXdbba9frFj0=OqFfea0dXdd9vqai=hGuQ8kuc9pgc9s8qqaq=dirpe0xb9q8qiLsFr0=vr0=vr0dc8meaabaqaciaacaGaaeqabaqabeGadaaakeaacqWGnbqtdaqhaaWcbaGaem4yamgabaGaemyAaKgaaaaa@30A6@

The number of times different texture profiles (*B*, *P*, *H*) were evaluated in classification varied according to a Poisson distribution controlled by *N *and the total number of samples in O
 MathType@MTEF@5@5@+=feaafiart1ev1aaatCvAUfKttLearuWrP9MDH5MBPbIqV92AaeXatLxBI9gBaebbnrfifHhDYfgasaacH8akY=wiFfYdH8Gipec8Eeeu0xXdbba9frFj0=OqFfea0dXdd9vqai=hGuQ8kuc9pgc9s8qqaq=dirpe0xb9q8qiLsFr0=vr0=vr0dc8meaabaqaciaacaGaaeqabaqabeGadaaakeaat0uy0HwzTfgDPnwy1egaryqtHrhAL1wy0L2yHvdaiqaacqWFoe=taaa@383C@_*n *_and O
 MathType@MTEF@5@5@+=feaafiart1ev1aaatCvAUfKttLearuWrP9MDH5MBPbIqV92AaeXatLxBI9gBaebbnrfifHhDYfgasaacH8akY=wiFfYdH8Gipec8Eeeu0xXdbba9frFj0=OqFfea0dXdd9vqai=hGuQ8kuc9pgc9s8qqaq=dirpe0xb9q8qiLsFr0=vr0=vr0dc8meaabaqaciaacaGaaeqabaqabeGadaaakeaat0uy0HwzTfgDPnwy1egaryqtHrhAL1wy0L2yHvdaiqaacqWFoe=taaa@383C@_*c*_. Thus, the number of repetitions *M *could be adjusted so that most of the samples were tested at least a predetermined number of times.

In order to refine the estimated log-likelihood ratios, we used a support vector regression algorithm operated by a radial basis function kernel [31-33] for an *ε*-insensitive cost function with *ε *= log(2
 MathType@MTEF@5@5@+=feaafiart1ev1aaatCvAUfKttLearuWrP9MDH5MBPbIqV92AaeXatLxBI9gBaebbnrfifHhDYfgasaacH8akY=wiFfYdH8Gipec8Eeeu0xXdbba9frFj0=OqFfea0dXdd9vqai=hGuQ8kuc9pgc9s8qqaq=dirpe0xb9q8qiLsFr0=vr0=vr0dc8meaabaqaciaacaGaaeqabaqabeGadaaakeaadaGcaaqaaiabikdaYaWcbeaaaaa@2DB9@). This final step ensured that the log-likelihood ratios varied smoothly across the texture feature profiles and substantially improved the reliability of the estimates.

**Figure 6:**
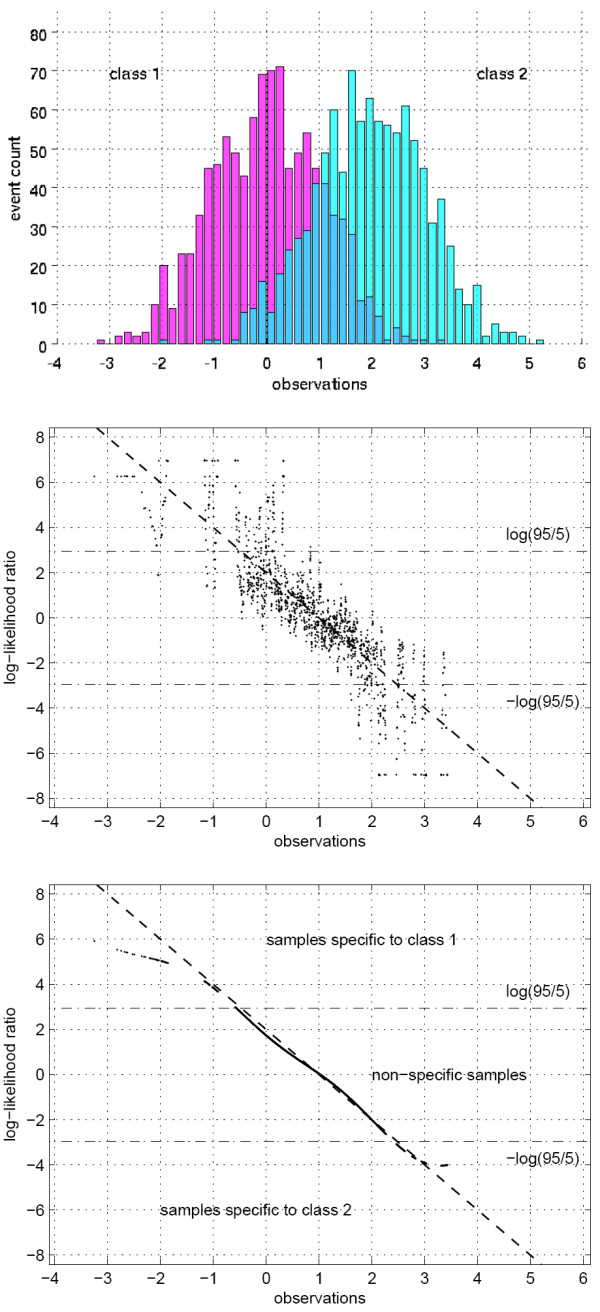
Statistical learning: Log-likelihood estimation procedure for unsupervised clustering of class-specific observations in a one-dimensional example. The histograms of two distinct classes of observations show substantial overlap between their distributions that are Gaussian with unit variance and means 0 and 2 respectively (top). The initial estimates of the log-likelihood ratio at the observations using the *k*-means strategy reveal the structure of the unknown true log likelihood ratio shown in the continuous line but are degraded by heavy noise (middle). The final estimates achieved using support vector machine regression accurately capture the unknown log-likelihood ratio and identify the samples that are specific to classes 1 and 2 along with those that are non-specific according to their log-likelihoods with respect to the 95% specificity thresholds given by ± log(95/5) (bottom).

The complete procedure used to estimate log-likelihood ratios at observed data points is illustrated in Figure 6. Two separate classes are shown with 1000 samples each, with Gaussian distributions at respective means 0 and 2 and unit variances. The procedure to estimate the log-likelihood ratios of the two classes at the observed samples repeated 1000 times provides noisy estimates, while the support vector machine regression estimate accurately captures the unknown true log-likelihood ratio. Note that only the samples over which at least one misclassification has been observed are included in the support vector regression procedure since the others do not carry any information on the log-likelihood ratios of the two classes at their specific locations in the observation space.

## 3 Results

This section presents our results on the segmentation of image blocks; distribution of texture parameters B, P, and H in the dataset of image blocks; the normal-specific, cancer-specific, and non-specific image block clusters; and their spatial distributions across histology slide images. Image regions that are comprised of cancer-specific blocks are considered as regions of interest and this information is utilized in sampling of the tumor tissue for constructing tissue microarrays with significant clinical relevance. Computations were carried out in parallel using grayscale and color tissue segmentation methods and results are presented for both segmentation methods.

### 3.1 Comparison of texture profiles via grayscale and color segmentation

**Figure 7:**
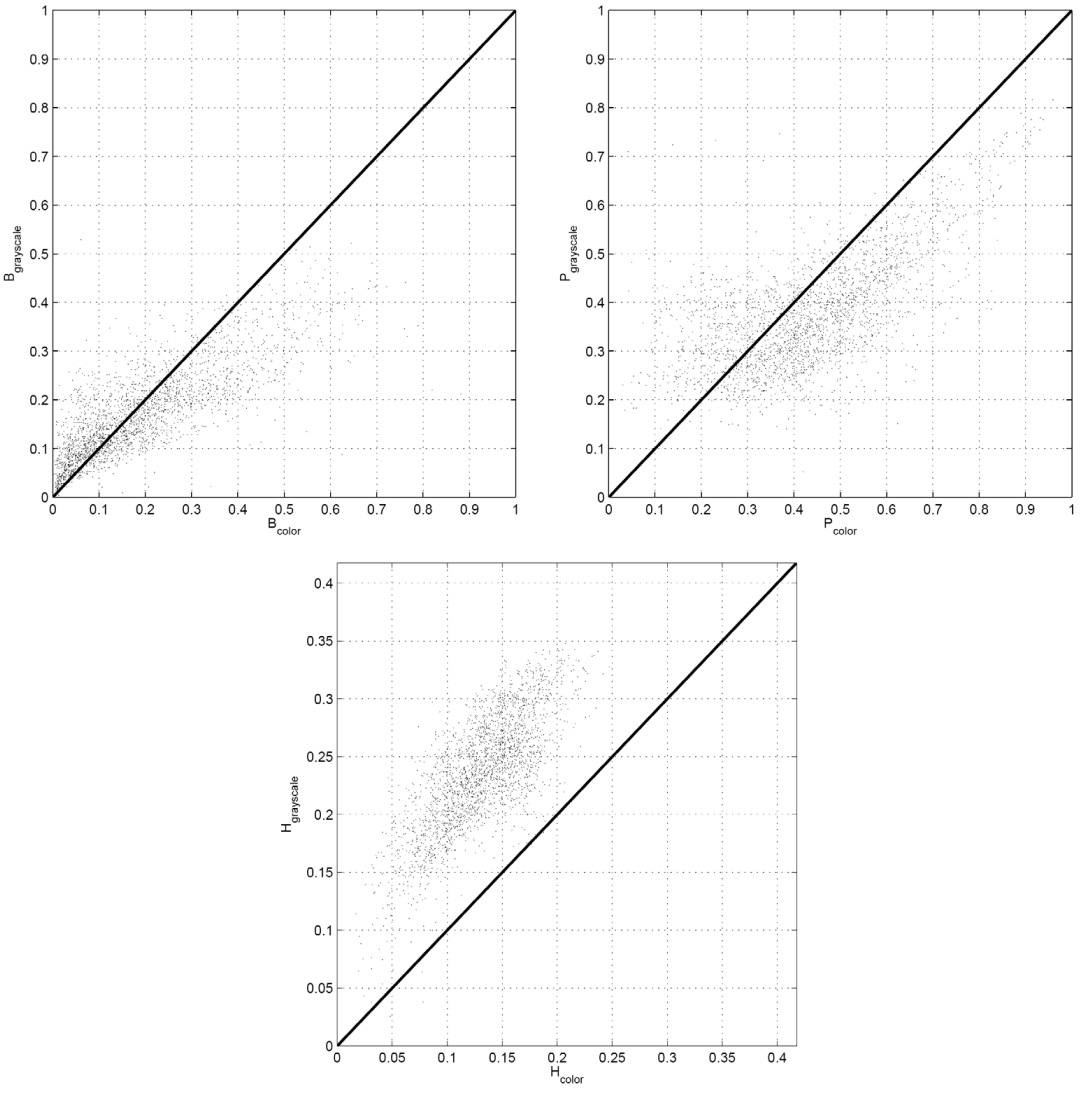
Comparison of the texture parameters obtained from histology image blocks using both grayscale and color tissue segmentation algorithms. The areas occupied by chromatin-rich (denoted by *B*) and stromal (denoted by *P*) regions as measured using grayscale and color segmentation are highly related through a non-linear mechanism though substantial deviation from the diagonal are also observed.

The grayscale tissue segmentation algorithms used in this study relies on the image intensities whereas the color tissue segmentation algorithms utilizes image luminance to identify the unstained regions first, and then uses image chromaticity indices to differentiate between the chromatin-rich and stromal regions. The examples in Figures 3 and 4 show that the tissue segmentation maps achieved by the two methods vary, and this variation is reflected on the texture parameters (*B*, *P*, and *H*) estimated for each image block using two different segmentation algorithms. Note that *B *and *P *represent the percentages of area of the image occupied by chromatin and stroma respectively, whereas *H *was defined in the Methods Section as a measure of heterogeneity in the image block. Scatter plots of *B*, *P*, and *H *obtained for each image block in the dataset algorithms are shown in Figure 7 for grayscale and color tissue segmentation. The figure indicates that the parameters *B*, *P*, and *H *vary significantly when computed by the two different segmentation methods for the same image block. For *B *and *P*, the relationship between the grayscale and color tissue segmentation measurements follows a nonlinear pattern. The grayscale segmentation algorithm provides larger values than the color segmentation when *B *and *P *are relatively low, and vice versa. This can be attributed to the preference of the *k*-means algorithm that forms the basis of grayscale segmentation to produce clusters of similar sizes, potentially leading to over-expressed values when they are low and under-expressed values when they are high. The scatter plot of the heterogeneity indices show a systematic difference between the two segmentation methods as *H *is estimated larger in grayscale segmentation than color segmentation. This can also be seen visually in the segmentation maps in Figures 3 and 4 where the map obtained by grayscale tissue segmentation is noticeably more grainy than the one obtained using color tissue segmentation, suggesting that color is more homogeneous across H&E stained tissue histology images than intensity. Since it is not possible to quantitatively determine which algorithm provides more accurate segmentation maps, we conducted the subsequent analysis using both segmentation algorithms in parallel. In the analysis of comprehensive image subsets involving different types of malignancy and/or tumors of different organs, the parameter set used in this article (*B*, *P*, *H*) can readily be revised and enriched with additional texture parameters causing minimal change in the rest of the log-likelihood estimation algorithm.

### 3.2 Detection of cancer-specific image blocks using statistical learning

**Figure 8:**
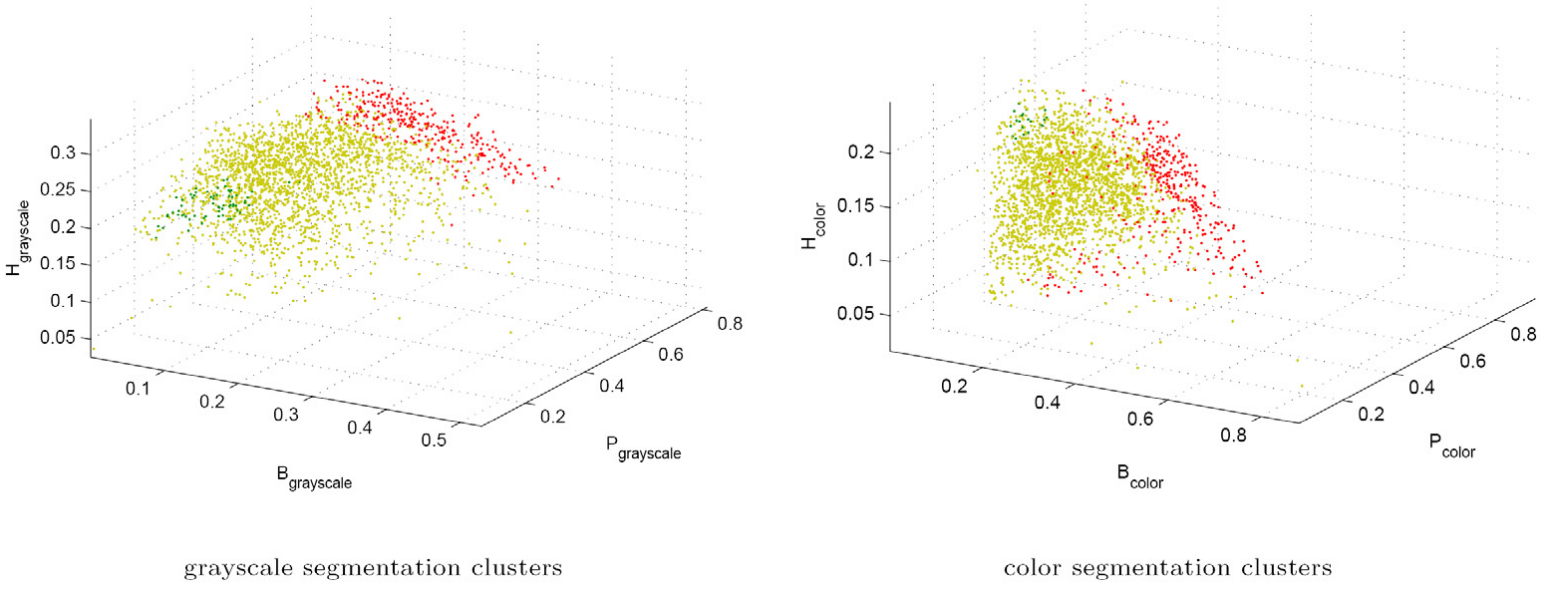
Clusters of texture appearance using parameters collected via grayscale and color tissue segmentation algorithms. Each image block is represented by the three texture parameters *B*, *P*, and *H*. The points colored green represent the image blocks that are specific to normal appearance, those colored red are those specific to cancer histology, and those colored yellow are those that are observed in both and thus are not specific to either.

We have determined the (*B*, *P*, *H*) profiles of the 2395 image blocks in our dataset. These texture features were then used as described in the Methods Section to classify the image blocks into three clusters: those that are specific to normal (**N**) and cancerous histology slides (**C**) and those that exhibit no particular specificity to cancer or normal tissue (**G**). The texture profiles of the normal-specific, cancer-specific, and non-specific image blocks as identified separately using grayscale and color tissue segmentation algorithms are shown in Figure 8. Not surprisingly, the bulk of the image blocks show no particular preference to either normal or cancerous histology slides, since a great portion of histology slides of cancerous samples are often occupied by normal appearing tissue around a cancerous neoplasm. By the same token, the number of image blocks that are specific to normal histology slides is very small (71 and 21 out of 2395 using grayscale and color segmentation respectively). This may be an artifact, or it may also signal the existence of certain histological appearances that disappear in highly invasive breast tumor tissue.

**Figure 9:**
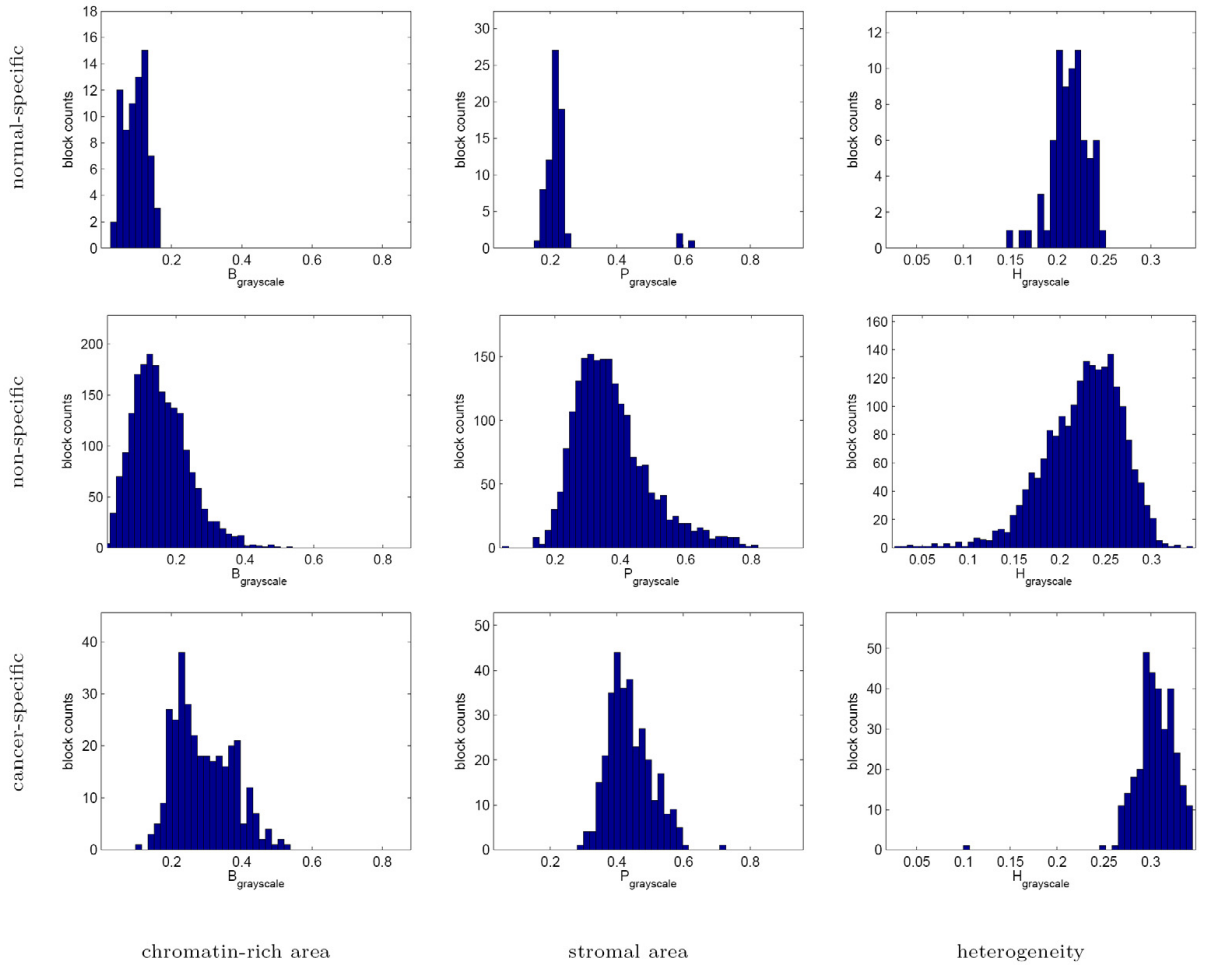
Texture profiles of the normal-specific, cancer-specific, and non-specific tissue block clusters as measured using grayscale tissue segmentation. Note that while the histograms of the individual parameters overlap between the three clusters, jointly they are significantly different and produce better than 95% specificity in normal-specific and cancer-specific clusters.

**Figure 10:**
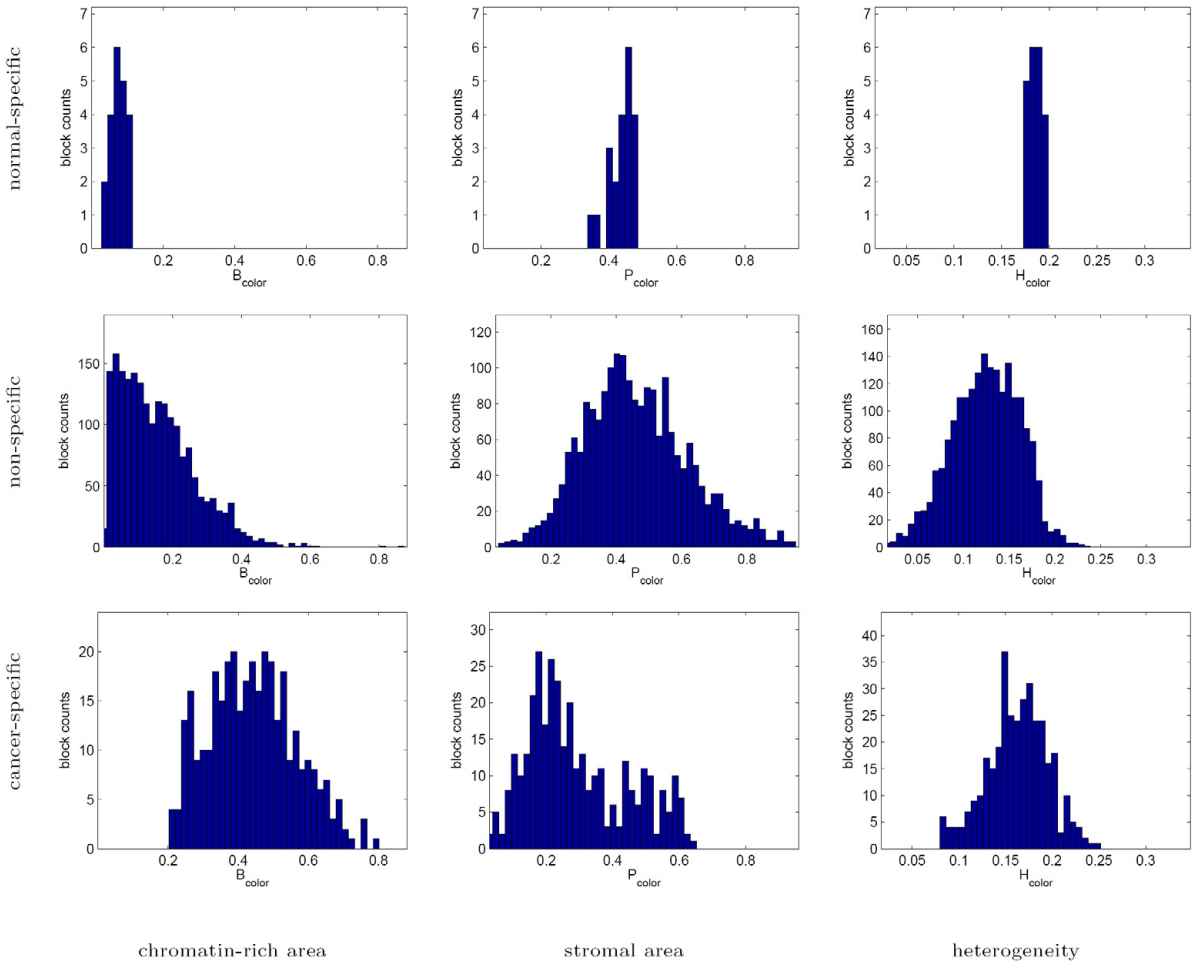
Texture profiles of the normal-specific, cancer-specific, and non-specific tissue block clusters as measured using color tissue segmentation. As before, while there is substantial overlap between the texture parameters of the three clusters individually, their joint analysis identifies the normal-specific and cancer-specific clusters with better than 95% specificity.

The histograms of texture parameters exhibited by the normal-specific, cancer-specific, and non-specific image block clusters in Figures 9 and 10 indicate that the cancer-specific cluster is commonly associated with highly chromatin-rich regions in both the grayscale and color tissue segmentations. The cancer-specific cluster is additionally characterized by increased heterogeneity in grayscale segmentation.

The confusion matrix that displays the overlap between the clusters identified based on grayscale tissue segmentation and color tissue segmentation algorithms in Table 1 indicate that even though the texture parameters collected from image blocks using the two schemes are different, the clusters with which they are identified are largely the same. This indicates that both segmentation methods have the potential to identify clinically relevant regions of interest on whole section histology images in an automated image analysis procedure.

**Table 1:** Confusion matrix of normal-specific, cancer-specific, and non-specific image block clusters obtained using grayscale and color tissue segmentation algorithms.

grayscale segmentation	color segmentation clusters			
	
Clusters	normal-specific	cancer-specific	non-specific	total
normal-specific	10	0	62	72
cancer-specific	0	226	94	320
non-specific	11	122	1870	2003

Total	21	348	2026	2395

**Figure 11:**
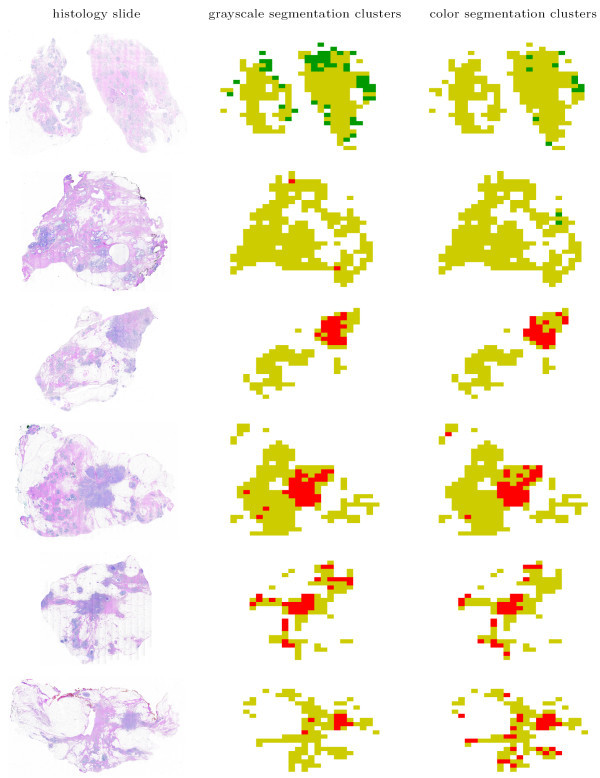
Identification of the normal-specific (shown in green), cancer-specific (shown in red), and non-specific (shown in yellow) appearance clusters on histology slides based on texture parameters computed using grayscale and color tissue segmentation algorithms. Relatively few image blocks are identified as normal specific (shown in green), while the cancer-specific image blocks conspicuously identify the tumors in the cancerous histology slides (shown in red). Overall, the delineations obtained by color tissue segmentation are more agreeable than those obtained using grayscale segmentation, as the latter misidentifies a few image blocks in normal histology slides as specific to cancer. Top two histology slides indicate normal tissue, whereas the bottom four have IDC, are shown at 1.25 times their actual size.

**Figure 12:**
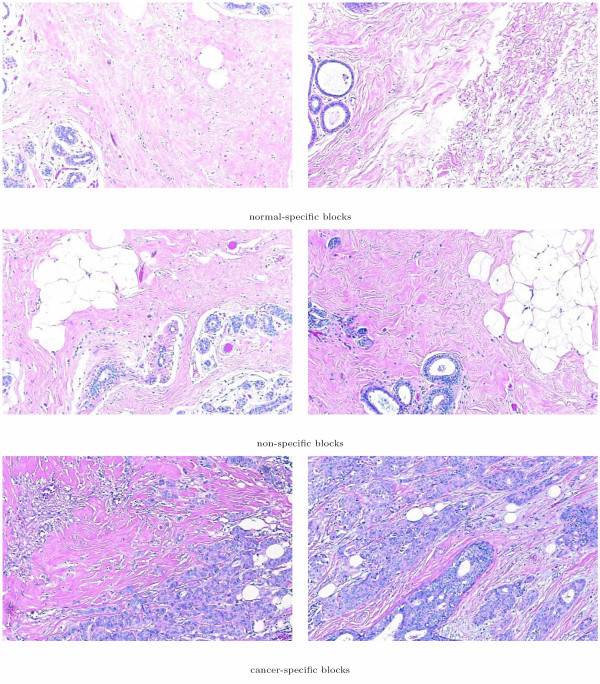
Sample image blocks of normal-specific, cancer-specific, and non-specific histological appearance clusters as identified simultaneously based on texture parameters obtained from grayscale and color tissue segmentation algorithms.

The spatial organization of the normal-specific, cancer-specific, and non-specific image blocks on several histology slides are shown in Figure 11. The figure shows that the images of whole section histology slides are dominated by image blocks in the non-specific cluster. The malignant neoplasms in the cancerous histology slides that were marked for us by the two pathologists mentioned in the Methods Section were identified as cancer-specific image blocks both by grayscale and color segmentation. Thus, the proposed methodology aptly recognized texture profiles that are not consistent with those observed in normal and benign breast tissue histology. Select examples of image blocks representing the three clusters commonly identified by both the analysis based on the grayscale tissue segmentation and that on color tissue segmentation are shown in Figure 12.

The composition of image block clusters obtained using grayscale and color tissue segmentation algorithms in terms of the image blocks of normal and cancerous histology slides computed in respective confusion matrices are shown in Tables 2 and 3. The 4 image blocks that are assigned to the cancer-specific cluster obtained using grayscale tissue segmentation algorithm in spite of having been observed in normal histology slides is within the 95% specificity criterion that has been employed to define the different clusters of histological appearance. The cluster assignments according to texture profiles obtained by color tissue segmentation are free from such misclassifications, suggesting that the color tissue segmentation algorithm may capture the texture features of image blocks more adequately than the grayscale tissue segmentation algorithm. While cancer-specific image blocks captured the malignant formations, the normal-specific blocks characterized tissue characteristics that are lost with the development of cancer.

**Table 2:** Confusion matrix between image block clusters identified following grayscale tissue segmentation and the diagnostic category of the histology slides of origin.

tissue block	histology slides of origin		
	
Clusters	normal	cancerous	total
normal-specific	72	0	72
cancer-specific	4	316	320
non-specific	1015	988	2003

Total	1091	1304	2395

**Table 3:** Confusion matrix between image block clusters identified following color tissue segmentation and the diagnostic category of the histology slides of origin.

tissue block	histology slides of origin		
	
Clusters	normal	cancerous	total
normal-specific	21	0	21
cancer-specific	0	348	348
non-specific	1070	956	2026

Total	1091	1304	2395

### 3.3 Guiding tissue sampling for tissue microarrays

High-density tissue microarray technology brings together rectangular tissue blocks from hundreds of different specimens [15,16]. While this allows examination of very large numbers of tissue blocks on a single slide, the degree at which the tissue blocks extracted from tumor tissue for production of tissue microarrays capture the full histological presentation of these tissues is not clear. In order to assess how completely the decisive histopathological features are represented by the selected tissue blocks, we have measured the classification composition (**N**, **C**, **G**) of square tissue blocks of approximate size 1 *cm*^2 ^placed at the center of mass of the histology specimen and at the center of mass of the regions that are compositionally indicative of cancerous appearance over the nine histology slides of IDC in our dataset. The composition of the selected tissue blocks in terms of non-specific, cancer-specific, and unstained regions as well as the coverage achieved of all cancer-specific regions on the histology slides are shown in Tables 4 and 5. For the purposes of this analysis, the cancer-specific regions were defined as those that are identified to be exclusive to cancerous appearance by either the grayscale tissue segmentation-based analysis or that based on color tissue segmentation.

**Table 4:** Composition of 9.86 × 9.27 *mm*^2 ^histology sections centered on the center of mass of the whole tissue slide in the nine cancerous histology slides in the dataset.

slide	tissue composition			
	
	non-specific tissue (%)	cancer-specific tissue (%)	unstained region (%)	cancer-specific tissue coverage (%)
1	21.48	9.63	68.89	39.39
2	36.30	26.67	37.04	90.00
3	34.07	36.30	29.63	39.52
4	18.52	11.85	69.63	100.00
5	37.78	36.30	25.93	96.08
6	51.85	28.15	20.00	80.85
7	29.63	20.74	49.63	48.28
8	25.93	16.30	57.78	56.41
9	38.52	12.59	48.89	56.67

mean	32.67	22.06	45.27	67.47
std	10.15	10.29	18.25	24.34

**Table 5:** Composition of 9.86 × 9.27 *mm*^2 ^histology sections centered on the center of mass of the cancer-specific regions in the nine cancerous histology slides in the dataset.

slide	tissue composition			
	
	non-specific tissue (%)	cancer-specific tissue (%)	unstained region (%)	cancer-specific tissue coverage (%)
1	11.11	24.44	64.44	100.00
2	22.22	28.89	48.89	97.50
3	34.07	38.52	27.41	41.94
4	15.56	11.85	72.59	100.00
5	37.78	36.30	25.93	96.08
6	37.04	31.85	31.11	91.49
7	25.93	22.22	51.85	51.72
8	22.96	15.56	61.48	53.85
9	40.74	17.04	42.22	76.67

mean	27.49	25.19	47.33	78.80
std	10.47	9.39	16.95	23.53

The results in Table 4 indicate that when the 1 *cm*^2 ^tissue block is selected arbitrarily at the center of mass of a full histology slide, only about 22% is occupied by cancer-specific regions on the average while almost half is occupied with unstained regions that are unworkable. Selecting the tissue block at the center of mass of cancer-specific regions significantly improves the average coverage of the cancer-specific regions, as shown in Table 5, but the coverage in cases where the malignancy appears spread out may still be poor, when only one or two blocks from the same tissue are included into the tissue microarray.

## 4 Discussion

This study presents a high throughput analysis of texture heterogeneity on breast tissue images for the purpose of identifying regions of interest in the tissue for molecular profiling via tissue microarray technology. Image texture was described in terms of three parameters: the percentage of area occupied in an image block by chromatin (*B*), percentage occupied by stroma like regions (*P*), and a statistical heterogeneity index *H *commonly used in image analysis. A typical whole section histology slide consisted of hundreds of image blocks comparable in size to tissue microarray spots. Texture parameters were defined and computed for each of the thousands of image blocks in our dataset using both gray scale and color segmentation. The image blocks were then classified into three categories using the texture feature parameters in a novel statistical learning algorithm. These categories are as follows: image blocks specific to normal breast tissue, blocks specific to cancerous tissue, and those that are non-specific to normal and disease states. Results indicated that both segmentation techniques were largely in agreement in classifying image blocks into the cancer-specific category. Moreover the image blocks identified as cancer-specific belonged to those cell crowded regions in whole section image slides that were already identified by pathologists as regions of interest for histological studies.

The statistical learning algorithm developed in this study was tested with success for three broad categories of texture images observed in normal or diseased breast tissue. Validity of our automated method of identification of cancer- and normal-specific tissue image textures is yet to be illustrated on a large set of images gathered in a clinical trial study. The method presented is a first step towards automated identification of clinically relevant image textures for cancer. It is expected that the method will require further refinement and improvement as it is challenged with tissue images gathered from a much larger pool of breast tumors that may contain images of a variety of non-neoplastic and pre-neoplastic conditions. Here, we have clearly demonstrated that given a set of learning texture images from histopathology, it is possible to recognize with very good accuracy similar textures in other histopathology images of breast tissue. Further improvements of the algorithm must include its adaptation to recognize texture images in a wide variety of tumor types. In the analysis of comprehensive image subsets involving different types of malignancy and/or tumors of different organs, the parameter set used in this article (B, P, H) can readily be revised and enriched with additional texture parameters causing minimal change in the rest of the log-likelihood estimation algorithm.

The automated texture image recognition algorithm developed for this article can readily be adapted to the recognition of additional histopathology textures. Incorporation of new data in the learning procedure is both possible and feasible: It only requires classifying them with respect to the reference sets used in randomized nearest neighbor classifications, and supplying the initial log-likelihood ratio estimates at the new data points to the support vector regression. As the amount of data incorporated into the system after the initial training grows large, a re-estimation of the log-likelihood ratios with new nearest neighbor reference sets may be performed to maintain maximum fidelity to all available data.

## 5 Conclusion

Results of this study indicate the high efficiency of our automated method for identifying pathologic regions of interest on histology slides. Automation of critical region identification will help minimize the inter-rater variability among different raters (pathologists) as hundreds of tumors that are used to develop an array have typically been evaluated (graded) by different pathologists. The region of interest information gathered from the whole section images will guide the excision of tissue for high throughput profiling of global gene expression. Recent studies by Schuetz et al. [34], Yang et al. [35], and Murphy et al. [36] indicate the importance of choosing the tissue sample for global gene expression profiling via image assessment of tissue texture. These studies utilize the laser capture microdissection tool to dissect regions of interest assessed by pathologists via microscopic examination. The procedure developed in this article would automate this process and eliminate possible human eye bias affecting the resultant data on the levels of activation of nearly 40000 genes.

Our method will play a similarly positive role in sampling tissue for tissue microarrays [16,37,38]. These tissue chips consist of paraffin blocks in which up to several thousand separate tissue cores are assembled in array fashion to allow simultaneous analysis of biomarker presence and absence as well as their spatial distribution. The recent advances in nanotechnology employing quantum dots allow multiple biomarkers to be shown on the images of the same array [39,40] and therefore there is a need for normalizing fluorescence image distribution with factors such as the parameter *B *of the present study indicating the extent of chromatin presence in the image. The technique proposed here can also be utilized as an initial screening phase of an automated image analysis for which the second phase focuses on more advanced techniques evaluating nuclei morphology [1,20-22,41] and/or the spatial arrangement of cell nuclei [23-25,42].

## Competing interests

The author(s) declare that they have no competing interests.

## Authors' contributions

Authors BK and AT designed the study and drafted the paper. Both authors have read and approved the final manuscript.

## Pre-publication history

The pre-publication history for this paper can be accessed here:


